# Trend of Conflict Rate in Iran from 2014 to 2020: An Ecological Study

**DOI:** 10.34172/aim.2023.23

**Published:** 2023-03-01

**Authors:** Elaheh Talebi-Ghane, Salman Khazaei, Fatemeh Hadavand Siri, Delniya Ahmadi, Ahmad Mehri

**Affiliations:** ^1^Modeling of Noncommunicable Diseases Research Center, Hamadan University of Medical Sciences, Hamadan, Iran; ^2^Health Sciences Research Center, Health Sciences & Technology Research Institute, Hamadan University of Medical Sciences, Hamadan, Iran; ^3^Department of Epidemiology, School of Public Health and Safety, Shahid Beheshti University of Medical Sciences, Tehran, Iran; ^4^Department of Epidemiology and Biostatistics, School of Health, Isfahan University of Medical Sciences, Isfahan, Iran

**Keywords:** Conflict rate, Growth mixture mode, Iran, Linear mixed effect model

## Abstract

**Background:**

Detecting the correlation of conflict rate within provinces over time provides a better understanding for health policymakers in identifying potential causes. The purpose of this study was to assess the trend of conflict rate in 31 provinces of Iran using the growth mixture model (GMM).

**Methods:**

This ecologic study was conducted based on the data obtained from the Iranian Legal Medicine Organization (ILMO) by gender and provinces between March 21, 2014 and March 21, 2020. First, the 7-year cumulative incidence rates were described; second, the trend of conflict rate was modeled by a linear mixed-effects model according to gender and overall; finally, distinct classes of provinces with similar conflict trends in seven years were identified using the GMM. The significance level was considered less than 0.05.

**Results:**

Among provinces, Ardebil and Sistan Baluchistan had the highest and the lowest 7-year conflict incidence rates (95% CI) with 66.6 (52.38, 84.67) and 20.79 (13.53, 31.95) per 100000, respectively. The results of the linear mixed-effects model showed that the annual rate of conflict in Iran decreased by 0.37% from 2014 to 2017 and then slightly increased by 0.07% after 2017. In addition, the GMM results indicated that the trends for Iranian provinces can be clustered into four distinct classes.

**Conclusion:**

Our study showed the increasing growth of conflict in the last years in most provinces of Iran. Necessary interventions are important to prevent the rising conflict rate due to the various effects of conflict on psychological, social, and health factors.

## Introduction

 Conflict is the quality of being likely to attack other people or animals or to behave in a violent or angry way towards them.^1^ Conflict behaviors, especially those that are self-abusing, are more common in people with anorexia nervosa and bulimia nervosa.^2^ This problem is both an individual issue and a range of social harm that affects public safety. There is no guarantee that conflict and violence will not result in beatings or even murder.^3^ Many crimes, such as street fighting, are carried out without prior planning and with the use of cold weapons, and after that, the defendants regret their actions.^4^

 There are various individual and community factors associated with conflict. Militant personality type, emotional poverty, failure, showing off, inappropriate socialization, lack of communication skills, and resorting to force to accomplish a right are some important person-related factors that can play a significant role in the occurrence of conflict.^5-8^ In terms of social factors related to conflict, issues such as lack of education, lack of appropriate patterns of leisure, unemployment or poor job condition, illiteracy or low level of education and lack of awareness of the negative consequences of conflict, low tolerance threshold individuals and inflamed social conditions in the community can lead to conflict.^9,10^ Moreover, living in marginalized and criminal areas, presence of violent groups in the form of thugs in the residential areas, and the acquaintance and entry of some young people into these groups are the main environmental factors related to conflict.^11-13^

 According to the World Health Organization, 1.6 million people worldwide die from violence and conflict. Violence and conflict are among the leading causes of death in the age of 15-44 years, especially in men.^14^ Another study shows that 25% of all women have been victims of violence and conflict for a lifetime duration. About 76% of detainees in African countries were imprisoned for domestic violence, sexual harassment, rape, and conflict in 2004.^15^

 Developed countries such as the United States, Canada, Sweden, and the United Kingdom also have the highest levels of violence after India, Afghanistan, Pakistan, and Sudan. The Gallup Institute also ranks Iran, Iraq, and South Sudan as the most controversial countries. The Gallup Thoughts Institute also named the Iranian people as one of the angriest people in 2018.^16^

 According to the statistics reported by the Iranian legal medicine organization (ILMO), more than half a million Iranian people injured in the conflict have referred to forensic medicine centers in 2017. An average of 1500 people come to these centers every day because of the conflict. About 30% of referrals to the ILMO are related to the conflict. This center also reported that 544 470 cases have referred to this center due to injuries caused by the conflict in 2018. The number of referrals to forensic medicine significantly increased to 580 070 cases in 2019, which shows an increase of 35 600 cases compared to the previous year.^17^

 Evaluation of conflict trends needs special techniques such as the linear mixed-effects model that can separate effects of province differences from time and also account for correlations of conflict rate within provinces over time. This model assumes that all provinces’ trends vary randomly around the overall mean trend and come from a single homogenous population.^18,19^ To determine exact variations among provinces trends and see whether Iranian provinces consist of mixture of distinct groups, growth mixture models (GMMs) are applied.

 Since no studies so far have investigated the trend of conflict rate in the provinces of Iran during the time, the findings of this study can be used by health and forensic epidemiologists, and mental health policymakers. Therefore, the purpose of this study was to describe and model the overall trend of conflict rate in Iran between 2014 and 2020 using a linear mixed-effects model, and finally, apply GMM to identify distinct classes of provinces with similar conflict trends over time.

## Material and Methods

 This ecologic study was conducted on data obtained from ILMO between March 21, 2014 and March 21, 2020. At the end of each year, ILMO publishes conflict cases reports on its website and access to this data is free for all.^20^ The people who have been injured by conflict referred to ILMO to determine their injury levels for legal action. Confirmation of conflict cases is based on autopsy evidence, examining the people’s bodies by a specialist, and an emergency medical report after being transferred to ILMO centers. This method of confirmation in all provinces is based on similar guidelines, and there is no difference in how to diagnose the conflict. These cases are classified based on the International Classification of Diseases, Eleventh Revision (ICD-11) code MB23.0,^21^ and verified by ILMO. The main data which was extracted from this website was the number of conflicts and population per 100 000 by gender for 31 provinces of Iran between March 21, 2014 and March 21, 2020. To obtain the rate of conflict, the number of conflicts was divided by the population in each year.

###  Statistical Analyses

 To assess the rates, firstly, descriptive statistics were reported by gender and province between 2014 and 2020 and then linear mixed random effects and GMM, as explained in Supplementary File 1, were applied. After fitting these models, the trends of weighted estimated averages of conflict rates were demonstrated in general and in each cluster. Moreover, the estimated average annual percent change in the rate of conflicts was presented within a map using ArcMap 10.8.All analyses were performed at 0.05 significance levels using Stata (Version 12.0) and Mplus (Version 6.0).

## Results

**Figure 1:**
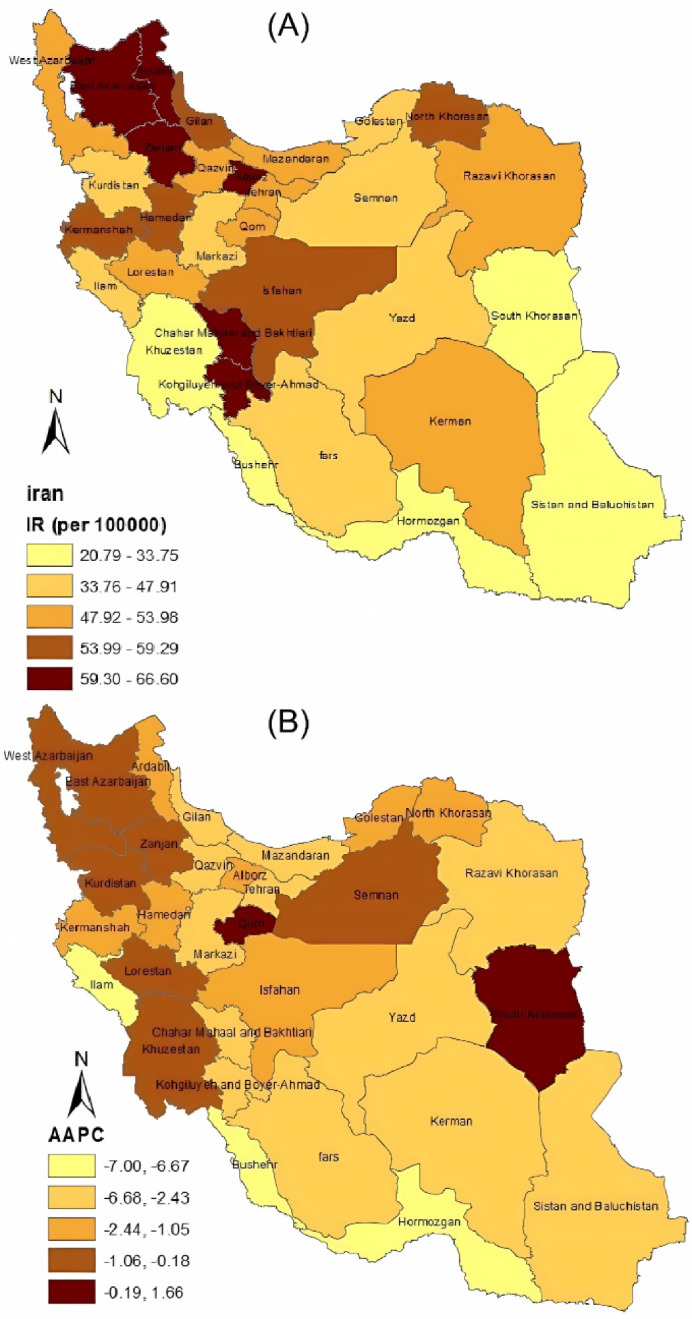
**(A)** 7-year incidence rate and **(B)** estimated average annual percent change in conflict rate for all provinces within a map

 Generally, the incidence rate of conflict (per 100 000 populations) for all 31 provinces of Iran was studied for seven years (2014–2020). The 7-year incidence rates of conflicts for all provinces are presented within a map in Figure 1A. Among these provinces, Ardebil and Sistan Baluchistan had the highest and the lowest 7-year incidence rates with 66.6 (52.38, 84.67) and 20.79 (13.53, 31.95) per 100 000, respectively. Table 1 shows the descriptive statistics for conflict rate in Iran by province and gender. As seen in this table, these rates were higher in males than females in all provinces. For males, the highest and the lowest mean conflict rates belonged to Ardebil and Hormozgan, respectively, whereas these rates belonged to Alborz and Sistan and Baluchistan provinces for females.

**Table 1:** Sorted 7-Year Incidence Rate of Conflict for Males, Females and Total

**ID**	**Male**		**Female**		**Total**	
	**Province**	**Incidence Rate** **(95% CI) Per 100000**	**Province**	**Incidence Rate** **(95% CI) Per 100000**	**Province**	**Incidence Rate** **(95% CI) Per 100000**
1	Ardebil	98.25 (80.63, 119.72)	Alborz	44.78 (33.41, 60.01)	Ardebil	66.60 (52.38, 84.67)
2	Chaharmahal and Bakhtiari	95.76 (78.39, 116.98)	Gilan	39.17 (28.64, 53.57)	Chaharmahal and Bakhtiari	64.96 (50.94, 82.84)
3	Kohgiluyeh and Boyer-Ahmad	90.79 (73.92, 111.51)	Razavi Khorasan	37.81 (27.49, 52)	Kohgiluyeh and Boyer-Ahmad	62.59 (48.86, 80.18)
4	East Azerbaijan	90.17 (73.36, 110.83)	Tehran	37.34 (27.1, 51.46)	East Azerbaijan	62.34 (48.64, 79.9)
5	Zanjan	87.29 (70.78, 107.65)	Qom	37.23 (27, 51.33)	Zanjan	61.60 (47.99,79.07)
6	Kermanshah	81.54 (65.64, 101.3)	Esfahan	36.95 (26.77, 51.01)	Alborz	61.25 (47.68, 78.67)
7	Hamadan	81.10 (65.24, 100.81)	North Khorasan	35.84 (25.84, 49.72)	Gilan	59.29 (45.97, 76.47)
8	Gilan	79.44 (63.76, 98.97)	Zanjan	35.46 (25.52, 49.28)	Kermanshah	58.42 (45.21, 75.49)
9	West Azerbaijan	78.17 (62.63, 97.56)	Kermanshah	34.78 (24.95, 48.49)	Hamadan	57.01 (43.98, 73.9)
10	North Khorasan	77.56 (62.09, 96.88)	Kerman	34.26 (24.51, 47.88)	North Khorasan	56.74 (43.74, 73.6)
11	Alborz	77.26 (61.82, 96.55)	Ghazvin	34.23 (24.49, 47.85)	Esfahan	55.66 (42.8, 72.38)
12	Lorestan	75.57 (60.32, 94.67)	Kohgiluyeh and Boyer-Ahmad	33.99 (24.29, 47.57)	Ghazvin	53.99 (41.35, 70.49)
13	Esfahan	73.81 (58.76, 92.72)	Ardebil	33.75 (24.09, 47.29)	Tehran	53.71 (41.11, 70.17)
14	Ghazvin	72.70 (57.77, 91.48)	East Azerbaijan	33.66 (24.01, 47.19)	Razavi Khorasan	53.43 (40.87, 69.86)
15	Tehran	69.86 (55.26, 88.31)	Chaharmahal and Bakhtiari	33.36 (23.76, 46.84)	West Azerbaijan	52.93 (40.43, 69.29)
16	Razavi Khorasan	68.87 (54.39, 87.21)	Mazandaran	33.24 (23.66, 46.7)	Qom	52.52 (40.08, 68.83)
17	Qom	67.25 (52.96, 85.4)	Hamadan	32.50 (23.05, 45.83)	Kerman	50.99 (38.75, 67.09)
18	Kerman	67.12 (52.84, 85.25)	Semnan	32.33 (22.9, 45.63)	Lorestan	50.93 (38.7, 67.02)
19	Mazandaran	65.64 (51.54, 83.6)	Yazd	32.23 (22.82, 45.52)	Mazandaran	49.55 (37.51, 65.45)
20	Golestan	65.27 (51.21, 83.18)	Markazi	30.87 (21.69, 43.93)	Markazi	47.91 (36.1, 63.59)
21	Kordestan	65.14 (51.1, 83.04)	Golestan	30.08 (21.04, 43)	Golestan	47.73 (35.94, 63.38)
22	Markazi	64.46 (50.5, 82.28)	Fars	28.75 (19.95, 41.43)	Yazd	46.57 (34.95, 62.06)
23	Ilam	61.58 (47.97, 79.05)	West Azerbaijan	26.99 (18.51, 39.36)	Semnan	46.32 (34.73, 61.77)
24	Yazd	60.09 (46.67, 77.37)	Lorestan	25.73 (17.48, 37.86)	Kordestan	45.09 (33.68, 60.37)
25	Semnan	59.91 (46.51, 77.17)	Kordestan	24.58 (16.55, 36.5)	Fars	43.80 (32.58, 58.89)
26	Fars	58.48 (45.26, 75.56)	South Khorasan	22.51 (14.89, 34.02)	Ilam	42.17 (31.19, 57.02)
27	Khuzestan	48.96 (37, 64.78)	Ilam	22.13 (14.59, 33.57)	Khuzestan	33.76 (24.1, 47.3)
28	Booshehr	38.44 (28.02, 52.73)	Khuzestan	18.16 (11.47, 28.76)	South Khorasan	29.60 (20.65, 42.43)
29	South Khorasan	36.50 (26.39, 50.48)	Booshehr	17.44 (10.91, 27.88)	Booshehr	28.77 (19.96, 41.46)
30	Sistan and Baluchistan	31.61 (22.31, 44.79)	Hormozgan	15.51 (9.43, 25.51)	Hormozgan	22.83 (15.15, 34.41)
31	Hormozgan	29.83 (20.84, 42.71)	Sistan and Baluchistan	9.79 (5.23, 18.32)	Sistan and Baluchistan	20.79 (13.53, 31.95)

**Figure 2:**
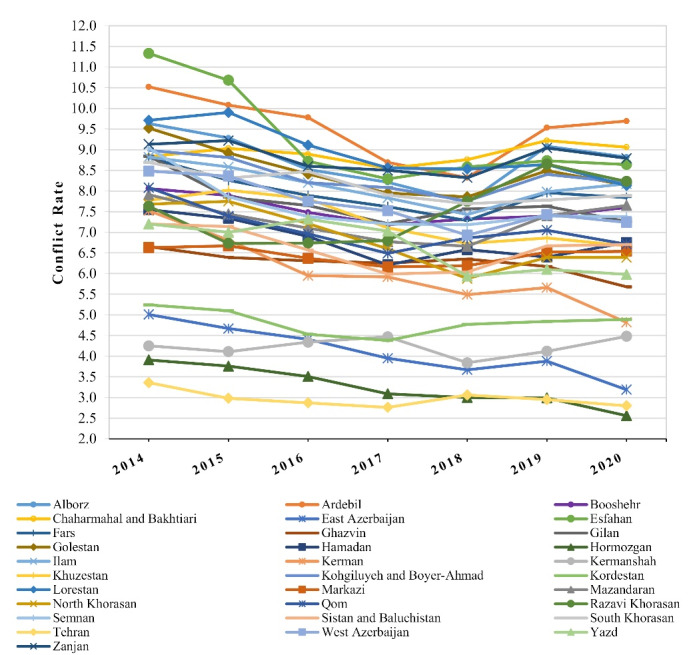
Trends of Conflict Rate for All Provinces in Iran During 2014–2020

 As preliminary analysis, the trend of the incidence rate of conflict for each province during the seven years is presented in Figure 2. A general gradual decrease was noticed in this figure during the seven years which was additionally confirmed in Figure 1B. This figure shows that the estimated annual change in rate within a map for all provinces is decreasing. It is observed inFigure 2 that there are different intercepts and growth slopes in conflict rates for the provinces under study. Therefore, a linear mixed-effects model was applied to evaluate the trend of the mean rate in these provinces from 2014 to 2020. Moreover, the weighted average of trends in Figure 2 showed a linear reduction from 2014 to 2017 and a slight linear growth until 2020. So, we assume a piecewise linear mixed-effects model with a knot in 2017. This model has an intercept and two slopes (one slope for changes in the mean rate before 2017, another for after 2017).

**Figure 3:**
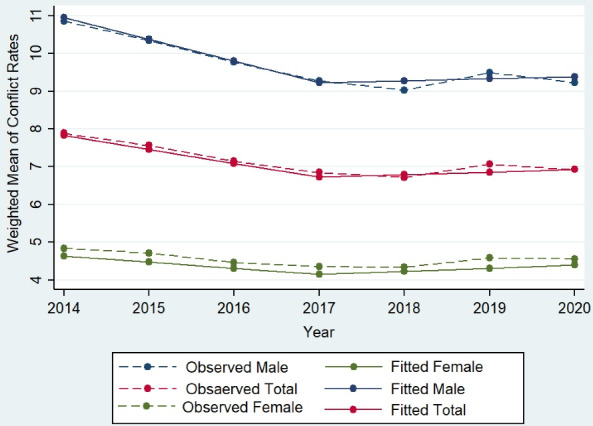
Weighted Estimated and Observed Trends of Conflict Rate in Iran by Gender During 2014–2020

 The results of the linear mixed effects model for estimating the trend of mean conflict rate during these seven years are presented in Table 2 and Figure 3. The estimated slopes for females, males and the total population indicate that the annual rates of conflict decreased by about 0.16%, 0.57% and 0.37%, respectively, before 2017 and increased approximately by 0.09% (sum of two estimates of time and (time)_ + _i.e. 0.25–0.16 = 0.09), 0.06% and 0.07% after 2017. Also, estimated variances of random terms show that there were fluctuations in provinces’ trends (intercept and slope) before and after 2017. Mean conflict rate patterns over time by gender are presented in Figure 3, separately for the observed and estimated mean of the random-effects model. As seen in this figure, the proximity of fitted and observed values shows the appropriate fitting of the random effects model to this dataset.

**Table 2:** Estimated Trend of Conflict Rate in All Provinces of Iran by Gender Using Linear Mixed-Effects Model

**Gender**	**Variable**	**Estimate**	**SE**	**95% CI**	***P *** **Value**
Female					
	Intercept	4.80	0.19	4.42, 5.18	< 0.001
	Time	-0.16	0.02	-0.20, -0.13	< 0.001
	(time)_ + _	0.25	0.03	0.19, 0.31	< 0.001
	Sigma intercept	1.05	0.14	0.82, 1.36	-*
	Sigma time	0.06	0.01	0.04, 0.09	< 0.001
	Sigma_(time)_ + _	0.09	0.03	0.05, 0.16	-*
	Sigma error	0.20	0.01	0.17, 0.22	-*
Male					
	Intercept	10.99	0.49	10.06, 11.48	< 0.001
	Time	-0.57	0.04	-0.66, -0.48	< 0.001
	(time)_ + _	0.63	0.07	0.49, 0.76	< 0.001
	Sigma intercept	2.68	0.35	2.07, 3.45	-*
	Sigma time	0.16	0.03	0.11, 0.24	< 0.001
	Sigma_(time)_ + _	0.18	0.07	0.08, 0.39	-*
	Sigma error	0.45	0.03	0.39, 0.52	-*
Total					
	Intercept	7.89	0.32	7.47, 8.73	< 0.001
	Time	-0.37	0.03	-0.43, -0.31	< 0.001
	(time) +	0.44	0.04	0.35, 0.52	< 0.001
	Sigma intercept	1.76	0.23	1.37, 2.27	-*
	Sigma time	0.10	0.02	0.07, 0.15	< 0.001
	Sigma_(time)_ + _	0.13	0.04	0.07, 0.25	-*
	Sigma error	0.29	0.02	0.25, 0.33	-*

*Could not be computed. SE, Standard error, sigma intercept, time and (time) + are the variance estimates for random effects of intercept and slopes (before 2017 and after 2017)

 The linear mixed-effects model assumes that all provinces come from a single homogenous population and their trends differ randomly around the overall mean; however, according to Figure 2, the existence of heterogeneity among provinces suggests that their trends should be categorized in different classes. Therefore, GMM was applied to identify distinct classes with similar trends over time.

 To cluster provinces’ trends according to the mean conflict rate, GMMs were fitted with 2 to 6 classes. Our results revealed that the 4-class GMM had the lowest Bayesian Information Criterion (BIC) (2457.85) and bootstrap likelihood ratio test (BLRT) for 5-class could not reject the 4-class model (*P* = 0.0865). Therefore, the conflict rate trend for these provinces were clustered into four classes: a linear growth model for class 2 and free time scores for others.

 The parameter estimates for 4-class GMM are shown in Table 3. Among 31 Iranian provinces, two provinces (6.5%) were classified in class 1, six provinces (18.9%) in class 2, seven provinces (24.5%) in class 3 and 16 provinces (50.1%) in class 4. The names of the provinces in each class are shown in this table alphabetically. Among these classes, class 2 had the lowest intercept and slope (5.09 and -0.17).

**Table 3:** Clustering of All Provinces Based on Conflict Rate Using Growth Mixture Model

**Parameter**	**CLASS 1** ^a^	**CLASS 2 ** ^b^	**CLASS 3 ** ^c^	**CLASS 4 ** ^d^
N (%)	2 (6.5%)	6 (18.9%)	7 (24.5%)	16 (50.1%)
Intercept	7.79*	4.49*	8.21*	8.22*
Slope	0.09*	-0.17*	-0.22	-0.38*
Time scores	(0, 1, 0.58, 0.68, 6.15, 11.51, 9.04)	(0, 1, 2, 3, 4, 5, 6)	(0, 1, 4.26, 6.16, 6.69, 5.93, 6.46)	(0, 1, 2.09, 3.06, 3.34, 1.89, 2.08)

Intercept represent the model estimated overall mean levels of initial and average rate of conflict change over time. Time score can be specified as linear for class II and free time scores for other classes. ^a^East Azerbaijan, Qom. ^b^Bushehr, Hormozgan, Ilam, Sistan and Baluchistan, South Khorasan, Yazd. ^c^Chahar Mahaal and Bakhtiari, Fars, Golestan, Kerman, Kohgiluyeh and Boyer-Ahmad, Markazi, Tehran. ^d^ Alborz, Ardabil, East Azerbaijan, Gilan, Hamadan, Isfahan, Kermanshah, Khuzestan, Kurdistan, Lorestan, Mazandaran, North Khorasan, Qazvin, Razavi Khorasan, Semnan, Zanjan.

**Figure 4:**
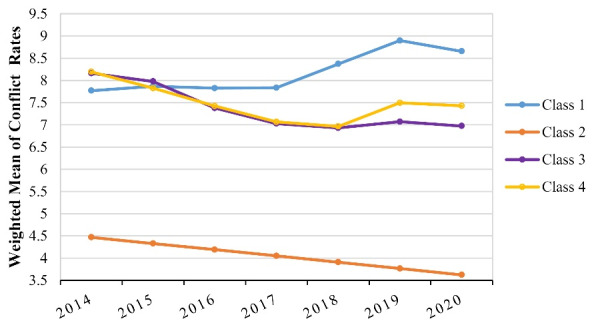
Weighted Estimated of Conflict Rate Trends for 31 Provinces Using GMM during 2014–2020

 As shown in Figure 4 and Table 3, class 2 had a linear trend and slope loadings (0, 1, 2, 3, 4, 5, 6), and others had nonlinear trends with free time score loadings. The conflict rate of provinces in class 1 can be defined as moderate at baseline (intercept_C1 _= 7.79) with a slow increase for three years (until 2017) which increased sharply until 2019 and since then declined gradually. Provinces in class 2 had the lowest intercept and decreasing slope (intercept_C2 _= 5.09, slope_C2 _= -0.17) and were defined as having a low conflict rate which persisted over time without a significant change.

 Provinces in classes 3 and 4 can be defined as having a high intercept at baseline (intercept_C3 _= 8.21 and intercept_C4 _= 8.22) which had an approximate sharp decrease until 2018. Since then, counties in class 4 had a gradual upward trend until 2019 and then declined slowly, while the countries in class 3 remained steady for two years. Notable about these two clusters is that the mean conflict rate of cluster 4 at the beginning of the study (2014) was the same as cluster 3 but at the end of the study (2020), this rate was higher than cluster 3.

 The obtained estimates in Table 3 can be interpreted regarding the specified free time scores. Interpretations for classes with the linear trend are more straightforward, but seem to be more complicated for classes with free time score loadings. For instance, the estimates for provinces with low conflict rate in class 2 (intercept = 4.49, slope = -0.17) show that the initial conflict rate in this cluster was 4.49 (in 2014) and the conflict rate has a decreasing trend with a slope of 0.17 from 2014 to 2020. Moreover, for provinces in class 4, the initial conflict rate was 8.22 in 2014 and estimated slope loadings from 2014 to 2020 were 0, 1, 2.09, 3.06, 3.34, 1.89, and 2.08, respectively; it can be demonstrated that the conflict rate had a decrease of -0.38 from 2014 to 2015 (1.0–0.0 = 1.0; slope_C4 _× 1.0 = -0.38), -0.41 from 2015 to 2016 (2.09–1.0 = 1.09; slope_C4 _× 1.09 = -0.41), -0.37 from 2016 to 2017 (3.06–2.09 = 0.97; slope_C4 _× 0.97 = -0.37), -0.11 from 2017 to 2018 (3.34–3.06 = 0.28; slope_C4 _× 0.28 = -0.11) and had an increase of 0.55 from 2018 to 2019 (1.89–3.34 = -1.45; slope_C4 _× -1.45 = 0.55). Finally, this rate declined to -0.07 between 2019 and 2020 (2.08–1.89 = 0.19; slope_C4 _× 0.19 = -0.07). The interpretations of estimates for other classes (classes 1 and 3) according to their estimated slope loadings are similar to class 4.

## Discussion

 This study aimed to investigate the trend of conflict rate in Iran from 2014 to 2020. Our results showed that the conflict rate decreased until 2017 and then showed a gradually increasing trend until 2020. This increase could reflect the diminishing resilience threshold and people’s tolerance, raising the number of conflict behaviors at the community level.^22^ Since the sanctions against Iran after 2017 have caused economic and social problems for the people,^23-25^ these events can be related to social issues,^26^ which may increase conflict. In support of this hypothesis, Kokabisaghi’s study^23^ shows that economic sanctions against the Iranian people can increase unemployment and inflation, reduce the people’s welfare, and ultimately lead to social problems such as violence and conflict. Other studies have also shown an association between socioeconomic problems and violence as a proxy of conflict.^27-29^ However, the quality of data collection in forensic centers and sensitivity of the system in the detection and reporting may change over the years and can justify some of the trend changes over the years.

 This study showed that the lowest conflict rate is related to the Sistan and Baluchistan province. Although underreporting and sensitivity of data recording may play a role in the status of statistics, one of the reasons for the low level of conflict can be due to ethnic leadership in this province. In this province, many people live in different tribes, which will lead to homogeneity of the population in terms of social issues.^30^ A study in Sistan and Baluchistan^31^ has shown that women continue to live with their abusive husbands despite being dissatisfied with their lives due to economic dependence on their husbands. Women also refrain from complaining to the judiciary and law enforcement in the face of violence because they are aware of patriarchal reactions. It seems that the tribal leaders and elders can help to prevent occurrence of conflict.

 As our findings showed, the highest conflict rate is related to the Ardabil province which was similar to the results of studies by Afshani et al^32^ and Mohammadoghli et al.^33^ Based on 30 articles related to conflict assessment in Ardabil, Javanmard et al showed that the tendency to conflict was moderate and high in 71% of people, which is consistent with our results.^20^ It seems that social factors affecting conflict formation can be the main reasons for the high level of conflict in this province. In support of this point, Keyanie and Fathi, in a study on adolescents in the Ardabil province, have shown that feelings of security, tolerance of distress, and social support were the predictors of conflict.^34^ In this section, attention must be paid to the sensitivity in reporting and recording data. However, further studies can be useful for finding the associated factors of conflict.

 This study showed that the provinces of Bushehr, Hormozgan, Ilam, Sistan and Baluchistan, South Khorasan, and Yazd had the lowest conflict rate compared to other provinces. After 2017, in half of the provinces, a gradual increase in conflict happened. Although there are no further studies to compare the results, the rising of conflict in provinces is a worrying indicator. Investigating the factors influencing the conflict and undertaking necessary interventions can prevent this increasing trend. Our study also showed that the conflict rate is higher in the northern and northwestern provinces and lower in the southern and southeastern provinces. The findings of this study are consistent with the fact that the prevalence of suicide and self-burning in the western and northwestern regions of Iran is higher than other regions, which is consistent with our findings in terms of the nature of the phenomenon.^35,36^ Consistent with this view, a study conducted in western Iran shows that self-burning in this region is directly related to violence.^37^ In addition, in the western and northwestern regions of Iran, urbanization is higher than other places, and as some studies have shown,^38-40^ the conflict, violence, and crime rate can increases with the rise in urban population. It seems that these problems are probably serious in this area and social, psychological and public interventions may be needed to solve problems such as conflict, violence and self-immolation.

 In terms of limitations, our study was based on data published by the Iranian Legal Medicine Organization (ILMO) and is an ecological study and thus, our results may be influenced by ecological fallacy. However, as various studies have shown, ecological studies are highly valuable on issues related to violence, crime and conflict.^41-43^ Further studies at the individual level and incorporating more variables can help identify the dimensions of conflict and show the epidemiological features of this issue. Moreover, in future studies, the interaction of time and provinces can be assessed. Our study is based on big data, and comparisons may be influenced by social differences, different cultures, and other social and demographic factors, which we could not adjust in the comparison between provinces. The coverage and the sensitivity of the reporting system are not the same among provinces, which can affect our results. However, since data entry in Iranian forensic centers is constantly reviewed and monitored, it seems that the contribution of sensitivity in data entry does not broadly affect our analysis. On the other hand, in this study, we examined the change in trends which can be more real. However, in the discussion section, these points were emphasized.

 In conclusion,assessing the time trend and clustering of Iranian provinces in terms of conflict were done for the first time in the current study which may form the basis for further studies. Our findings showed changes in an important social and health indicator. Based on the current study, an increasing growth of conflict rate was observed over the last years in Iran. Necessary interventions are important to prevent the rising conflict rate due to the various effects of conflict on psychological, social, and health factors. Conducting further epidemiological and population-based studies can be essential to identify the factors related to the conflict.

## Supplementary File

Supplementary file 1. Linear mixed effects model.
