# Supplementary material for: Trend of Conflict Rate in Iran from 2014 to 2020: An Ecological Study
Source: Arch Iran Med. 2023 Mar 1;26(3):147–55. doi: 10.34172/aim.2023.23 (PMC10685728; doi:10.34172/aim.2023.23)
Supplement: Supplementary file 1 — Linear mixed effects model. [file aim-26-147-s001.pdf]

Arch Iran Med. March 2023;26(3):147-155
10.34172/aim.2023.23
http://journalaim.com

Supplementary file 1

Linear mixed effects model

As preliminary analyses showed, there are different intercepts and growth slopes in conflict rates for the provinces under study. Therefore, a linear mixed effects model was applied to evaluate the trend of mean rate in these provinces from 2014 to 2020. This trend has a linear reduction from 2014 to 2017 and a slight linear growth until 2020 (Figure 1). So, we assume a piecewise linear mixed effects model with a knot in 2017. This model has an intercept and two slopes (one slope for changes in the mean rate before 2017, another for after 2017) which can be specified as:

$$E(Y_{ij}|b_i) = \beta_1 + \beta_2 time_{ij} + \beta_3 (time_{ij})_+ + b_{1i} + b_{2i} time_{ij} + b_{2i} (time_{ij})_+$$

Where $time_{ij}$ denotes year of jth measurement on the ith province before or after 2017, $(time_{ij})_+ = time_{ij}$ if $time_{ij}>2017$ and $(time_{ij})_+ = 0$ if $time_{ij} \leq 2017$. $(\beta_1 + b_{1i})$ is intercept for ith province, $(\beta_2 + b_{2i})$ and $\{(\beta_2 + \beta_3) + (b_{2i} + b_{3i})\}$ are the ith province's slope before and after 2017 (18, 19)

Growth Mixture Model (GMM)

$$y_{it}^k = \eta_{i0}^k + \eta_{i1}^k \lambda_t^k + \varepsilon_{it}^k$$

$$\eta_{i0}^k = \eta_{00}^k + \sum_j \beta_{01j}^k x_j + \varsigma_{i0}^k$$

$$\eta_{i1}^k = \eta_{10}^k + \sum_j \beta_{11j}^k x_j + \varsigma_{i1}^k$$

Where y_{it}^k is conflict rate for the ith province at time t; η_{i0}^k and η_{i1}^k are latent variables, λ_t^k is time score which can be specified as linear, nonlinear polynomial functions of time or free time

scores; ε_{it}^{k} is residual term for ith country at time t; η_{00}^{k} and η_{10}^{k} are intercept coefficients representing the model estimated overall mean levels of initial and average rate of conflict change over time; β_{01j}^{k} and β_{11j}^{k} are slope coefficients of covariates x_j; ς_{i0}^{k} and ς_{i1}^{k} are error terms (23, 24).
